# Compliance with diabetes guidelines at a regional hospital in KwaZulu-Natal, South Africa

**DOI:** 10.4102/phcfm.v5i1.447

**Published:** 2013-04-08

**Authors:** Okoroma J. Igbojiaku, Ogbonnaya C. Harbor, Andrew Ross

**Affiliations:** 1 Department of Family Medicine, Howard Campus, University of KwaZulu-Natal, South Africa; 2 Department of Family Medicine Ngwelezane Hospital, KwaZulu-Natal, South Africa

## Abstract

**Background:**

Diabetes is a major problem in South Africa and throughout the world. The management of type 2 diabetes aims at maintaining normoglycaemia and preventing the development of complications arising from diabetes. The Society for Endocrine Metabolism and Diabetes of South Africa (SEMDSA) guidelines are based on a number of international trials which showed that strict control of blood sugar leads to a reduction in the development of diabetic complications. However, many studies have shown poor adherence to national guidelines by doctors caring for diabetes patients.

**Objectives:**

The aim of this study was to assess doctors’ compliance with the SEMDSA diabetes guidelines at a regional hospital in KwaZulu-Natal.

**Method:**

Seven hundred and fifty diabetic patient records were selected by systematic sampling of cases from the diabetic clinic and reviewed against SEMDSA guidelines.

**Results:**

Eighty three per cent of the patients had high values of glycated haemoglobin (HbA_1c_). Lipid examination was rarely performed, and comprehensive foot examination was carried out in only 6% of patients. Although blood pressure and weight were regularly checked, these examinations were performed by the nursing staff, and medical staff generally did not respond to abnormal results.

**Conclusion:**

This study demonstrates poor compliance with current diabetic guidelines. There is an urgent need to review how guidelines are disseminated and implemented in South African public sector hospitals if evidence-based guidelines are to have any impact on patient care.

## Abstrait

**La conformité avec les indications nationales à une clinique de diabète d'un hôpital régional dans KwaZulu-Natal; Afrique du Sud**

**Fond:**

Le diabète est un problème majeur à Afrique du Sud et à travers le monde. La direction de type 2 objectifs de diabète à maintenir de normoglycaemia et empêcher le développement de complications résultant du diabète. La Société pour le Métabolisme et Diabète Endocrine d'Afrique du Sud (SEMDSA) les indications sont fondées sur un nombre de procès internationaux qui ont montré ce contrôle strict de glucide mene à une réduction dans le développement de complications diabétiques. Cependant beaucoup d’études ont montré l'adhésion pauvre aux indications nationales par les médecins qui soignent les malades de diabète.

**Objectifs:**

L'objectif de cette étude était d’évaluer de la conformité des médecins avec les indications de diabète de SEMDSA à un hôpital régional dans Kwa Natal zoulou.

**Méthode:**

Sept cent et cinquante dossiers patient diabétiques ont été choisis par l’échantillonnage systématique de cas de la clinique diabétique et ont été réexaminés contre les indications de SEMDSA.

**Résultats:**

Quatre-vingt trois pourcent des malades a eu haut des valeurs de glycated haemoglobin (HbA_1c_). L'examen de lipide a été rarement fait, l'examen de pied complet a été seulement fait pour six pourcent de malades. Bien que la tension artérielle et le poids ont été régulièrement vérifiés, ces examens ont été faits par le personnel qui soigne et le personnel médical n'a pas répondu généralement aux résultats anormaux.

**Conclusion:**

Cette étude a démontré la conformité pauvre avec les indications diabétiques actuelles. Il y a un besoin urgent de réexaminer comment les indications sont disséminées et sont exécutées dans les hôpitaux de secteur publics, africains et du sud si la preuve a basé des indications sont d'avoir n'importe quel impact sur soins aux patients.

## Introduction

With over 285 million people with diabetes worldwide, there is no doubt that this disease is a major health problem throughout the world. Diabetes is the fourth leading cause of death in the Western world and the leading cause of chronic renal failure.^1^ Diabetes also significantly contributes to morbidity and mortality associated with ischaemic heart disease and cerebrovascular accidents.

Diabetes is not only a disease of affluent countries – increased urbanisation, with a more westernised diet and sedentary lifestyle, has led to a large number of patients developing diabetes in developing countries. It is estimated that in 2010 there were 4 million people with diabetes in South Africa, equating to a prevalence of 4.5% of the general population.^1^

### Key focus

The benefits of good glycaemic control were demonstrated by two major studies: the Diabetic Control and Complication Trial and the United Kingdom Prospective Diabetes study performed in 2003. Both studies concluded that development of complications in diabetic patients was directly related to their glycaemic level.^2, 3^ In a nine-year follow-up study involving 4662 men and 5570 women, Kay-Tee Khaw (2003, cited in Hereyan, 2004)^4^ demonstrated that a 1% rise in the level of glycated haemoglobin (HbA_1c_) was associated with an increased risk of death of up to 28% and 24% in women and men respectively. HbA_1c_ levels below 5% were associated with lowest rates of cardiac complications and death. However, such levels were also associated with significant hypoglycaemia.^5^

In a 2004 study Klausen et al.^6^ showed that the presence of micro-albuminuria was a predictor of early renal pathology, and levels > 4.8 µg/min were strongly associated with coronary heart disease and death from cardiac events. Based on international research findings, the Society for Endocrine, Metabolism and Diabetes of South Africa (SEMDSA) developed guidelines for the management of diabetic patients in South Africa, which were updated in 2009. The guidelines set a target for HbA_1c_ of < 7% for all diabetic patients, as well as specific targets for lipids, blood pressure, body mass index and when various monitoring assessments should be conducted. Regular monitoring and appropriate response to abnormal results have been shown to reduce complications such as myocardial infarction, cerebrovascular accidents and retinopathy in diabetic patients.^5^

### Background

Adherence to diabetic guidelines when managing diabetic patients has been shown not only to reduce complications but also to improve utilisation of resources. In 1995 a study was performed in South Africa amongst specialist physicians and general practitioners with an interest in diabetes management who had been trained in the American Diabetes Association Guidelines targets. In this study doctors received financial incentives to adhere to the guidelines. Improved diabetes management based on the guidelines resulted in a 90% reduction in hospitalisation amongst diabetic patients, demonstrating conclusively that with incentives and motivation adherence to guidelines was possible, and that such adherence to guidelines benefits patients.^7^ A number of studies have reported innovative ways to remind doctors to follow guidelines, including reminders by nurses, computer prompts and use of standardised recording sheets which show which investigation needs to be performed and when. Under research conditions all of these methods have been shown to be useful in improving adherence to guidelines.^8, 9^

### Trends

However, many studies have shown that doctors in general do not adhere to guidelines. A study carried out in Norway in 1997 which reviewed patient data retrospectively to evaluate adherence to National Diabetic Guidelines by general practitioners concluded that doctors were not adhering to the guidelines. Analysis by the Centers for Disease Control and Prevention found that < 5% of diabetic patients in the United States of America received care equivalent to what is specified in the American Diabetes Association guidelines.^8, 10^

### Objectives

The aim of this study was to assess doctors’ compliance with the core process of care as stated in the SEMDSA diabetes guidelines, and also to review demographic and clinical profiles of diabetic patients at a regional hospital in KwaZulu-Natal.

### Contribution to the field

The SEMDSA guidelines have been widely distributed in South Africa and are available for those running diabetic services throughout the country. To date there has not been an evaluation of adherence of doctors to the SEMDSA diabetic guidelines at Ngwelezane Hospital, and this study aims to address this gap.

## Ethical considerations

Permission to conduct this study was obtained from the Research and Ethics Committee of the University of KwaZulu-Natal (Ref. No. BE165/010), the provincial Department of Health and hospital management.

### Potential benefits and hazards

This study highlights the need for improve adherence to guidelines amongst primary healthcare providers to help reduce complications of diabetes. There was no direct hazard since the study took the form of a data review without human participants. The data were only accessible to the researcher and remained locked in a safe place, where they will be for years.

## Trustworthiness

### Reliability

The data collection material was taken directly from the SEMDSA guidelines.

### Validity

This was a descriptive study and hence may not be generalised outside of its context; also, this study was not able to consider all possible confounders.

## Methods

### Materials

The checklists in the patient records that were assessed in this study included the following: patient demographic data and HbA_1c_, lipid profile, blood pressure, weight and/or body mass index, waist circumference, comprehensive foot examination, micro-albuminuria, serum creatinine, eye examination, referral to dietician and diabetic nurse educator. They also state how many times these processes of care were carried out, and their values.

### Setting

The study was performed at a large regional hospital 180 km north of Durban between June and October 2011. The diabetic clinic sees 2600 patients per year and receives referrals from 16 clinics.

### Design

A study sample of 750 records was chosen, representing 30% of the 2600 patients with type 2 diabetes who are seen annually at the clinic. Inclusion criteria were regular attendance at the diabetic clinic for > 1 year and type 2 diabetes. Patients with type 1 diabetes (defined as onset of diabetes prior to 40 years of age and presentation with ketonuria) and patients with gestational diabetes were excluded from the study.

### Procedure

Records were assessed against the SEMDSA guidelines. A random number between one and three was chosen and then every third record was chosen until 750 charts had been selected.^11^

### Analysing

Data were captured onto an Excel spreadsheet and analysed using the Statistical Package for Social Sciences. Descriptive analysis such as mean, median, mode and interquartile range were use to summarise the data, and results were presented in tables and graphs.

## Results

Of the 750 files reviewed, 514 were on females (68.5%) and 236 on males (31.5%). The mean age was 53 years (40–90 years), 82% were unemployed, 95.6% (717 out of 750) were Black people, 2.7% (20 out of 750) were White people and 1.7% (13 out of 750) were Indian people. The average number of visits to the diabetic clinic was 5 per year (2 to 10 visits).

Only 24% (180 out of 750) of the patients had their HbA_1c_ checked in the preceding year (Table 1 [a–b]). Of the 180 who had their HbA_1c_ checked, only 16.7% (30 out of 180) had values within target (< 7%). Twenty two patients had the HbA_1c_ check repeated, of whom only two had normal values. Only two patients had their HbA_1c_ checked on more than two occasions, and both had values higher than the target.

**TABLE 1a:** Frequency of HbA_1c_ when assessed for the first time and at quarterly visits.

Valid	*f*	%
Performed	180	24
Not performed	570	76

**Total**	**750**	**100**

*f*, frequency.

**TABLE 1b:** HbA_1c_ analysis at quarterly clinic visits.

Valid	*N*	Minimum (%)	Maximum (%)	Mean (%)	sd
HbA_1c_ 1	180	5.4	15.4	9.7	2.4206
HbA_1c_ 2	22	6.4	12	8.9	1.6886
HbA_1c_ 3	2	8	8.4	8.2	0.2828

sd, Standard deviation;%, percentage; *n*, number of patients with HbA_1c_ performed; HbA_1c_. 1, 2, 3, quarterly values.

Five hundred and fifty six (74%) patients had total cholesterol checked at least once. The mean value was 4.9 mmol/L (2 mmol/L-9 mmol/L) (Table 2 [a–c]). Of those who had their cholesterol measured, 44.2% (246 out of 556) had normal values of total cholesterol. No patient had their low-density lipoprotein (LDL) or high-density lipoprotein (HDL) measured. Triglycerides were recorded as assessed in 295 (39.3%) of patients, of whom 43.1% (127 out of 295) had values within the target of < 1.7 mmol/L.

**TABLE 2a:** Lipid profile frequency.

Valid	*f*	%	Valid (%)	Cumulative (%)
Performed	556	74.1	74.1	74.1
Not performed	194	25.9	25.9	100

**Total**	**750**	**100**	**100**	-

*f*, frequency;%, percentage.

**TABLE 2b:** Quarterly lipid profile and analysis.

Valid	*f*	%	Valid (%)	Cumulative (%)
1	344	45.9	61.9	61.9
2	170	22.7	30.6	92.4
3	42	5.6	7.6	100

**Total**	**556**	**74.1**	**100**	-

*f*, frequency.

**TABLE 2c:** Quarterly lipid profile and analysis.

Valid	*f*	%
Not performed	194	25.9
	750	100

*f*, frequency.

All patients had their blood pressure measured on at least three occasions during the year (Table 3 [a–b]), and the vast majority of patients had their blood pressure checked on each visit. Of the 750 patients only 39.6% (297 out of 750) had a systolic blood pressure at or below the target of 130 mmHg, and only 38.7% (290 out of 750) a diastolic blood pressure at or below the target of 80 mmHg. Seven hundred and thirty three patients had their weight recorded at least once, and the mean weight was 86 kg (45 kg – 153 kg). Ninety nine per cent of patients did not have any record of body mass index, and waist circumference was not recorded in 97.6% (732 out of 750) of files. Only 6.1% (46 out of 750) of files had documented evidence of foot examination having been performed, and of the 46 patients who did have their feet examined, nine had abnormal findings.

**TABLE 3a:** Blood pressure values at six visits to diabetic clinic.

*n*	SBP 1	DBP 1	SBP 2	DBP 2	SBP 3	DBP 3
Valid	750	750	750	750	750	750
Not Performed	0	0	0	0	0	0
Median	136	84	137	83	136	82
Mode	114	70	132†	70	130	84
Minimum	90	43	87	42	87	49
Maximum	246	133	234	140	228	180

SBP, systolic blood pressure; DBP, diastolic blood pressure.

Multiple modes exist; the lowest value is shown.

† Multiple modes exist. The smallest value is shown.

*n*, number of patients with Blood pressure performed.

**TABLE 3b:** Blood pressure values at six visits to diabetic clinic.

*n*	SBP 4	DBP 4	SBP 5	DBP 5	SBP 6	DBP 6
Valid	749	749	718	718	688	687
Not performed	1	1	32	32	62	63
Median	136	82	141	82	140	82
Mode	132	70	120†	70	142	70†
Minimum	60	45	60	42	68	48
Maximum	215	182	255	181	1161	147

Multiple modes exist; the lowest value is shown.

SBP, systolic blood pressure; DBP, diastolic blood pressure.

† Multiple modes exist. The smallest value is shown.

*n*, number of patients with Blood pressure performed.

**FIGURE 1:**
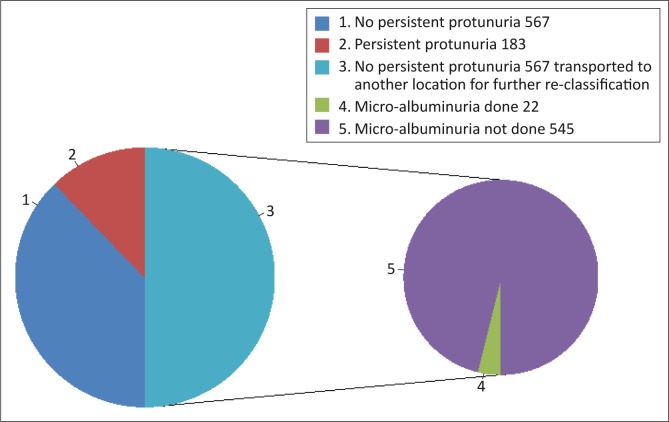
Presence of persistent proteinura and micro-albuminuria.

Urine dipstick testing was performed in all patients, of whom 24.4% (183 out of 750) had persistent proteinuria. Of the 567 patients without proteinuria (Figure 1), only 3.9% (22 out of 567) had their urine tested for micro-albuminuria, and five of these had abnormal results.

Eighty-seven per cent of patients had their blood tested for serum creatinine. The hospital has a functional ophthalmology department, and 43% of the patients (323 out of 750) had at least one eye examination in the preceding year. Of the 323 patients who had an eye examination, cataracts were diagnosed in 15 patients, diabetic retinopathy in 62 patients, and two patients were blind.

The SEMDSA guidelines recommend referral of diabetic patients to a nurse educator to reinforce diabetic education, and to a dietitian to help patients make appropriate modifications to their eating habits. However, there was a poor record of referral to the nurse educator and only 120 patients were referred to the dietitian.

## Discussion

The results of this study are disappointing, considering that the SEMDSA guidelines have been widely distributed and are freely available, and that this study was performed at a diabetic clinic in a regional hospital. The results show poor compliance with processes of care (when to do which investigations), and poor patient outcomes as shown by the large numbers with elevated HbA_1c_ levels, abnormal lipid results, poorly controlled blood pressure, proteinuria and eye abnormalities.

In terms of outcome-related results, these finding are similar to the baseline finding of a study performed at the Pretoria Academic Hospital in 2004:^9^ a baseline mean HbA_1c_ of 9.77 in the Pretoria study compared to 9.708 in our study. However, our study differs from the Pretoria study in that only 6% of patients had a foot examination in this study, compared to 23.4% of patients in Pretoria prior to the intervention, but is similar to the 4.7% found in a Cape Town study.^9, 12^

In terms of process-related results, the total cholesterol was measured in 74.1% of patients, which compares very favourably with the baseline results in the Pretoria study, which found that only 20.6% of patients had their cholesterol checked prior to their intervention. HDL and LDL were not assessed for any of the 750 patients in our study, despite the guideline recommending that HDL, LDL, total cholesterol and triglycerides be measured at least twice a year. Other studies have only reported lipid profile, which is often not checked at the prescribed intervals.^9, 12^

This study has confirmed what other studies have shown – that processes of care are performed well when they are part of routine care and part of someone's day-to-day responsibility.^7^ Blood pressure and urine dipstick assessments carried out by nursing staff were well recorded in the notes. However, patients with no proteinuria, who needed to have their urine assessed for micro-albuminuria, were not well assessed. A standing order for nursing staff to test urine for micro-albuminuria if the urine is negative for protein may be a way to improve compliance in this area.

There has been much discussion recently in South Africa about the need to re-engineer primary health care. A key component of the re-engineering document to ensure adequate human resources for the provision of health care is task shifting, where routine tasks are delegated to the lowest level of competency.^13^ The development of mid-level workers, currently being trained at the Universities of Pretoria, Witwatersrand and Walter Sisulu, could provide the opportunity to delegate many of these processes of care to competent healthcare.

However, abnormal results need to be acted upon if patient care is to improve, and simply task shifting the responsibility to ensure that tests are performed and blood pressures are checked will not improve patient outcomes. Acting on abnormal results must be the responsibility of the doctor caring for the patient. The finding that 60% of patients had abnormal blood pressure readings suggests that doctors are either not recognising abnormal results or are not responding appropriately to abnormal results – both of which potentially lead to poor outcomes and development of complications in patients.^2, 3^

The findings of this study concur with a number of other South African and international studies which have shown that overall management of diabetic patients, knowledge of patients about their illness and management of the diabetic foot are suboptimal.^12, 14^ A study in Norway amongst primary healthcare providers found a low level of adherence to diabetic guidelines, despite substantial investment in circulating guidelines and educating doctors on them.^10^

Recommendations from the study in Pretoria^9^ which showed improvement in processes and outcome of care for diabetic patients include use of a structured consultation schedule and a programme of continuing medical education for those involved in care of diabetic patients. There is, however, no evidence that benefits gained during the study period were sustained once the study was finished. Other studies have recommended organisational support and computer-tracking systems to help doctor implement guidelines.^8^

South Africa is known for a progressive Constitution, great legislation and well thought out policies; however, poor implementation of many of these has impacted negatively on service delivery to those who need it most.^15^ There is a danger that the healthcare profession will end up in the same trap – great evidence-based guidelines, unless consistently implemented, will have no impact on improving the quality of care provided to patients.

## Limitations of the study

The major limitation to this study was time constraints, which limited the number of records that we could assess.

## Conclusions and recommendations

This study has highlighted poor processes of care and poor patient outcomes at a regional hospital with a dedicated diabetic referral clinic. It highlights the need to review how guidelines are distributed and implemented if patient management and outcomes are to be improved. Recommendations from this study include the following:

- The need to assess knowledge of common guidelines as an exit competency when students leave medical school.
- The need to teach and assess ability of healthcare professionals to update their knowledge of common guidelines.
- Development of a culture of excellence where reviews, audits and quality improvement projects are part of the standard operating system within all units.
- Institutionalising quality improvement initiatives, where regular audit of the processes and outcomes of care by those involved in patient care are monitored.
- Conducting regular continuing medical education sessions which focus on evidence-based management of diabetic patients.
- Carrying out more research on innovative ways to ensure that guidelines are implemented.
